# Thymol Nanoemulsion: A New Therapeutic Option for Extensively Drug Resistant Foodborne Pathogens

**DOI:** 10.3390/antibiotics10010025

**Published:** 2020-12-30

**Authors:** Mahmoud M. Bendary, Doaa Ibrahim, Rasha A. Mosbah, Farag Mosallam, Wael A. H. Hegazy, Naglaa F. S. Awad, Walaa A. Alshareef, Suliman Y. Alomar, Sawsan A. Zaitone, Marwa I. Abd El-Hamid

**Affiliations:** 1 Department of Microbiology and Immunology, Faculty of Pharmacy, Port Said University, Port Said 42511, Egypt; 2 Department of Nutrition and Clinical Nutrition, Faculty of Veterinary Medicine, Zagazig University, Zagazig 44511, Egypt; doibrahim@vet.zu.edu.eg; 3 Infection Control Unit, Zagazig University Hospital, Zagazig 44511, Egypt; rashamosbah@hotmail.com; 4 Drug Radiation Research Department, National Center for Radiation Research and Technology (NCRRT), Atomic Energy Authority, Cairo 11865, Egypt; farag3m2012@gmail.com; 5 Department of Microbiology and Immunology, Faculty of Pharmacy, Zagazig University, Zagazig 44511, Egypt; waelmhegazy@daad-alumni.de; 6 Department of Avian and Rabbit Medicine, Faculty of Veterinary Medicine, Zagazig University, Zagazig 44511, Egypt; nf2731982@gmail.com; 7 Department of Microbiology and Immunology, Faculty of Pharmacy, October 6 University, 6th of October 12566, Egypt; Dr.walaa@o6u.edu.eg; 8 Doping Research Chair, Department of Zoology, College of Science, King Saud University, Riyadh 11495, Saudi Arabia; syalomar@ksu.edu.sa; 9 Department of Pharmacology and Toxicology, Faculty of Pharmacy, Suez Canal University, Ismalia 41522, Egypt; Sawsan_zaytoon@pharm.suez.edu.eg; 10 Department of Pharmacology and Toxicology, Faculty of Pharmacy, University of Tabuk, Tabuk 71491, Saudi Arabia; 11 Department of Microbiology, Faculty of Veterinary Medicine, Zagazig University, Zagazig 44511, Egypt; mero_micro2006@yahoo.com

**Keywords:** thymol nanoemulsion, XDR, *Salmonella* Enteritidis, broiler chickens, cytokines

## Abstract

Foodborne pathogens have been associated with severe and complicated diseases. Therefore, these types of infections are a concern for public health officials and food and dairy industries. Regarding the wide-spread multidrug resistant (MDR) and extensively drug resistant (XDR) foodborne pathogens such as *Salmonella* Enteritidis (*S.* Enteritidis), new and alternative therapeutic approaches are urgently needed. Therefore, we investigated the antimicrobial, anti-virulence, and immunostimulant activities of a stable formulation of thymol as thymol nanoemulsion in an in vivo approach. Notably, treatment with 2.25% thymol nanoemulsion led to a pronounced improvement in the body weight gain and feed conversion ratio in addition to decreases in the severity of clinical findings and mortality percentages of challenged chickens with XDR *S.* Enteritidis confirming its pronounced antimicrobial activities. Moreover, thymol nanoemulsion, at this dose, had protective effects through up-regulation of the protective cytokines and down-regulation of XDR *S.* Enteritidis *sopB* virulence gene and interleukins (*IL*)*-4* and *IL-10* cytokines as those hinder the host defenses. Furthermore, it enhanced the growth of gut *Bifidobacteria* species, which increases the strength of the immune system. For that, we suggested the therapeutic use of thymol nanoemulsion against resistant foodborne pathogens. Finally, we recommended the use of 2.25% thymol nanoemulsion as a feed additive for immunocompromised individuals as well as in the veterinary fields.

## 1. Introduction

Recently, one of the most important current crisis and threats to public health is the infection from resistant foodborne pathogens. Unfortunately, high morbidity and mortality rates were observed among human, animals, and poultry infected with multidrug-resistant (MDR) and extensively drug resistant (XDR) bacteria because of limited therapeutic options [1]. The extensive spread of MDR foodborne pathogens has created unexpected treatment failure [2]. Despite the development of novel drugs, the MDR foodborne pathogens are predominately problematic owing to the dramatic increase in the number of infected patients and the acquisition of resistance genetic elements among those bacteria [3]. *Salmonella* Enteritidis, *Staphylococcus aureus*, *Campylobacter jejuni,* and *Listeria monocytogenes* are the most common poultry and human infections, which cause foodborne diseases and represent high risks to both human and poultry health [4,5,6,7]. Many life threating diseases are caused by MDR and XDR *Salmonella* Enteritidis, which has the ability to express a large number of virulence factors, especially Salmonella outer proteins (Sops) and *Salmonella* Enteritidis fimbrial (sefA) protein [8]. 

Prevention and fighting of salmonellosis start with increasing the strength of the immune system. Cytokines are important proteins that act as communication signals between cells to protect against the enteric infections [9]. Multiple immunomodulators and proinflammatory cytokines have protective roles against salmonella infections such as interleukins (IL)-1alpha, tumor necrosis factor (TNF) alpha, interferon gamma (IFN-γ), IL-12, IL-18, and IL-15. Meanwhile, other cytokines like IL-4 and IL-10 inhibit the host defenses against salmonella infections [10]. It is essential to find an alternative therapy such as essential oils (EOs) to fight the resistant *Salmonella* Enteritidis foodborne pathogen and prevent its virulence [11]. Extensive documentation ensured the antimicrobial properties of thymol essential oil and its mechanisms of action in order to control the MDR bacterial infections [12]. Additionally, thymol could be used in poultry nutrition as a feed additive as it improved growth performance parameters and feed efficiency of poultry through enhancing the digestibility. From the viewpoint of cost-effectiveness, the minimum inhibitory concentration (MIC) values of most essential oils are significantly higher than the recommended dose in animal production [13], especially thymol oil, which is completely absorbed in the stomach and the proximal small intestine after oral administration. However, it has low water solubility, which reduces its biological activity and limits its application [14]. Additionally, it has low physical and chemical stability in the presence of oxygen, light, and temperature, which reduces its efficiency [15]. These problems might be canceled by preparation of thymol nanoemulsions [16]. Thymol nanoemulsion has high physical and chemical stability in the aqueous medium [14]. Moreover, this formulation allows control of the release of active ingredients on the target site and reduces their volatility and protects them from interaction with the environment [17].

As the problem of resistant foodborne pathogens grows, the infection with MDR strains will be replaced by XDR pathogens. Therefore, we postulated the wide spread of XDR foodborne pathogens globally in the near future. Thymol essential oil was pronounced to be one of the most therapeutic options, but its activity against XDR pathogens remains variable and it is affected by its physical form. For that, the development of new formulations of thymol oil to fight these types of infections caused by XDR foodborne pathogens is pivotal and it still has the advantages of the alternative therapy in contrast to chemical antimicrobial agents. Therefore, this experimental study aimed to assess the antimicrobial, anti-virulence, immunostimulant activities of thymol nanoemulsion and to determine the most effective concentration against XDR *Salmonella* Enteritidis infection among differently challenged broiler chickens.

## 2. Results

### 2.1. Characterization and Antibiogram of MDR and XDR S. Enteritidis Strains

**Figure 1:**
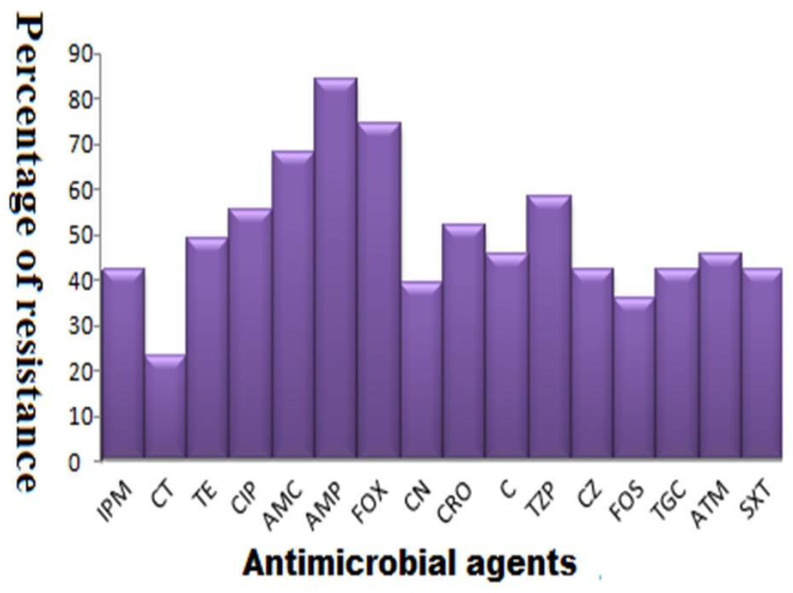
Antimicrobial susceptibility patterns of *S.* Enteritidis strains. IPM: imipenem, CT: colistin sulfate, TE: tetracycline, CIP: ciprofloxacin, AMC: amoxicillin/clavulanic acid, AMP: ampicillin, FOX: cefoxitin, CN: gentamicin, CRO: ceftriaxone, C: chloramphenicol, TZP: piperacillin/tazobactam, CZ: cefazoline, FOS: fosfomycin, TGC: tigecycline, ATM: aztreonam, SXT: sulfamethoxazole/trimethoprim.

Thirty one *S.* Enteritidis strains were identified by standard microbiological techniques and they were then confirmed by the serotyping technique and genetic detection of *sefA* gene. According to an antimicrobial susceptibility test, the lowest resistance rates were observed against colistin sulfate and fosfomycin (22.6% and 35.5%, respectively). Meanwhile, the highest resistance rates were recorded against ampicillin and cefoxitin (83.9% and 74.2%, respectively) as shown in Figure 1. Although the high rate of MDR pattern (83.9%, 26/31), which was observed among our strains, all the investigated *S.* Enteritidis strains exhibited absolute sensitivity to at least three antimicrobial categories with the exception of one human isolate, which was identified as an XDR strain. Fortunately, the XDR strain was still sensitive to colistin sulfate and fosfomycin.

### 2.2. In Vitro Antibacterial Activities of Thymol Nanoemulsion

The minimum inhibitory concentration (MIC) and the minimum bactericidal concentration (MBC) values of thymol nanoemulsion against identified MDR and XDR *S.* Enteritidis strains were determined (Table S1). The MIC values of thymol nanoemulsion against MDR and XDR *S*. Enteritidis strains were 0.5–1 and 3%, respectively. Moreover, the MBC values against MDR and XDR *S.* Enteritidis strains were 1–2 and 5%, respectively. Accordingly, we selected thymol nanoemulsion 0.25, 0.5, 0.75, and 1 MIC values, which were equivalent to 0.75, 1.5, 2.25, and 3% to be used later in our in vivo experiment for the treatment of birds challenged with the identified XDR *S.* Enteritidis strain.

### 2.3. Ultrastructure of Treated S. Enteritidis Strains Using Transmission Electron Microscope (TEM)

**Figure 2:**
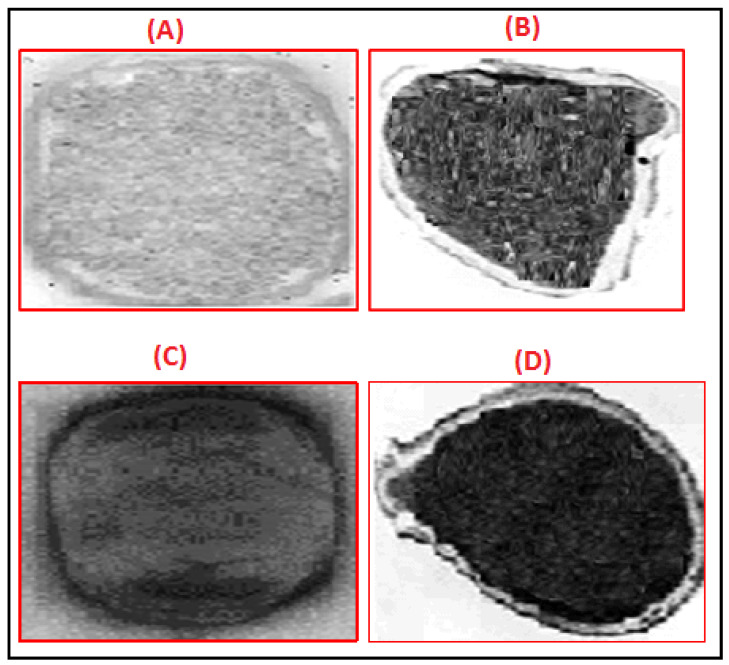
Transmission electron micrographs of untreated and thymol nanoemulsion treated *S.* Enteritidis. (**A**,**C**) are untreated multidrug resistant (MDR) and extensively drug resistant (XDR) strains with complete cell walls and cell structures. Meanwhile, (**B**,**D**) are MDR and XDR strains with damaged cell membranes and cell walls with complete cell destruction after exposure to MBC doses of thymol nanoemulsion.

Transmission electron microscope was used to elucidate the antibacterial mechanism of action of thymol nanoemulsion. As shown in Figure 2, the treatment of MDR and XDR strains with MBC doses of thymol nanoemulsion led to the complete destruction of the bacterial cells. Thickening and damage of both cell wall and cell membrane in addition to cytoplasmic leakage and clumping were also observed. This confirmed the powerful antibacterial activities of thymol nanoemulsion.

### 2.4. In Vivo Antibacterial, Anti-Virulence, and Immunostimulant Activities of Thymol Nanoemulsion

#### 2.4.1. Clinical Examination

The clinical signs recorded in broiler chickens post *S.* Enteritidis challenge were ruffled feathers, loss of appetite, weakness, depression, poor growth, diarrhea, pasty vent, dehydration, and thirst. The postmortem examination of freshly dead or sacrificed birds revealed gross lesions in the form of hepatomegaly with necrosis, enlarged spleen, pericarditis, perihepatitis, and enteritis with necrotic lesions in the mucosa. The challenged non-treated (positive control) group exhibited sever degrees of the previously described clinical and postmortem findings. Mild degrees of clinical signs and postmortem lesions were noticed in the four challenged and thymol nanoemulsion treated groups without a detectable difference between the higher doses (2.25 and 3%). Meanwhile, no clinical signs appeared in the unchallenged and untreated (negative control) group.

#### 2.4.2. Growth Performance

Growth performance parameters of broiler chickens throughout the experimental period are shown in Table 1. Compared with the negative control group, the challenged non-treated group had significantly decreased final body weight and body weight gain and increased the feed conversion ratio (*p* < 0.0001). Treatment with thymol nanoemulsion in the challenged groups resulted in a highly significant improvement in both body weight gain and feed conversion ratio (*p* < 0.0001), which were ameliorated in the chicken groups treated with 2.25 and 3% thymol nanoemulsion.

#### 2.4.3. Mortality Rates

Although, the high mortality rate observed in the positive control group (22%), significant decreases in mortality percentages were observed in challenged chicken groups treated with thymol nanoemulsion, especially at its 0.75 and 1 MIC values (2.25 and 3%) (*p* < 0.0001) (Table 1).

#### 2.4.4. Effect of Thymol Nanoemulsion on Gut Microbiota Counts

**Figure 3:**
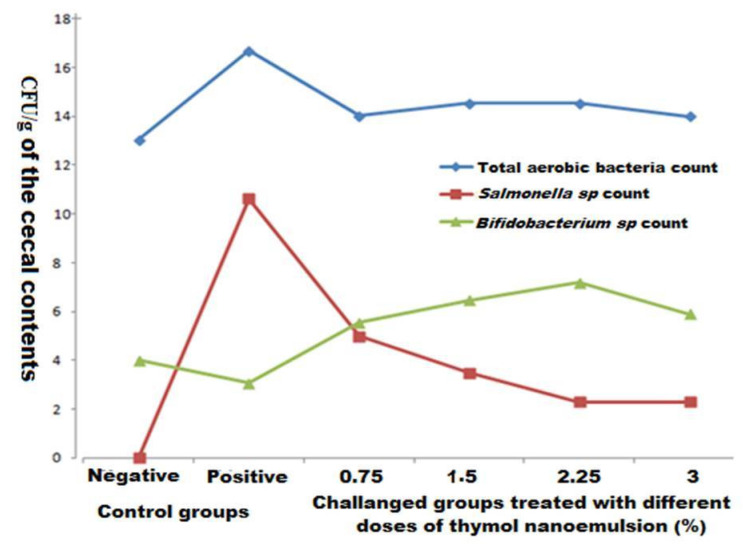
Bacterial counts in cecal contents of *S.* Enteritidis challenged broilers as affected by treatment with 0.75, 1.5, 2.25, and 3% thymol nanoemulsion.

The total bacterial counts increased in the challenged non treated (positive control) group compared to the unchallenged and untreated (negative control) one as shown in Figure 3. Furthermore, a significant rise in the numbers of total bacterial counts were determined in cecal contents of all treated groups compared with the negative control group (*p* < 0.0001), which reached the maximum levels in the challenged groups treated with 1.5 and 2.25% thymol nanoemulsion. The numbers of *Bifidobacterium* species increased significantly in all treated groups compared with the positive and negative control groups (*p* < 0.0001). This increase was dose dependent, except at 3% thymol nanoemulsion. Additionally, lower significant levels of salmonella populations were detected in all treated groups with respect to the positive control group (*p* < 0.0001). The maximum reduction levels of salmonella counts were observed among the challenged groups treated with 2.25 and 3% thymol nanoemulsion. This was confirmed by measuring the DNA copies of *S.* Enteritidis *sefA* gene. Interestingly, *S.* Enteritidis counts were reduced in response to thymol nanoemulsion treatment in a dose-dependent manner and reached to a steady state among the birds treated with 2.25 and 3% thymol nanoemulsion when compared to the positive control group.

#### 2.4.5. Anti-Virulence Activity of Thymol Nanoemulsion

**Figure 4:**
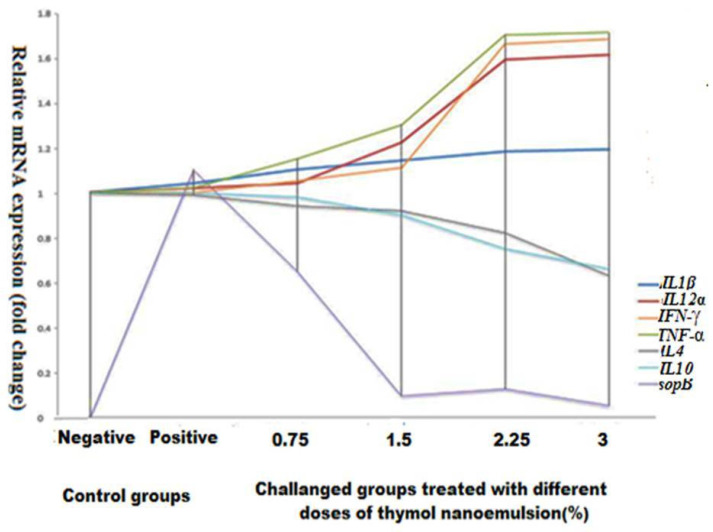
Expression of cytokines and *sopB* genes in the splenic and cecal samples of *S.* Enteritidis challenged broilers in response to treatment with 0.75, 1.5, 2.25, and 3% thymol nanoemulsion, respectively.

The expression of *sopB* virulence gene in all challenged groups was measured by quantitative reverse transcription PCR (RT-qPCR). Surprisingly, the expression levels of *sopB* gene were significantly decreased in all treated groups compared to the positive control group (*p* < 0.0001) with a sharp decrease in the challenged groups treated with 1.5% thymol nanoemulsion (0.05-fold change), which reflects the anti-virulence activity of thymol nanoemulsion against XDR *S.* Enteritidis (Figure 4).

#### 2.4.6. Gene Expression Analysis of Cytokines

There was a significant up regulation in the expression of *IL1β*, *IL12α*, *IFN-γ*, and *TNF-α* genes (*p* < 0.001) in all treated groups in comparison with the negative control group (up to 1.66-fold change) in a dose-dependent manner. Of note, the increase in the cytokine genes expression levels nearly reached a steady state in the challenged groups treated with 2.25 and 3% thymol nanoemulsion. In another context, in a dose-dependent manner, the expression of *IL-4* and *IL-10* genes was significantly (*p* < 0.0001) decreased (up to 0.63-fold change) as shown in Figure 4.

## 3. Discussion

The emerging and increasing prevalence of resistance to multiple antimicrobial agents among pathogenic bacteria [18,19,20] and fungi [21] has become a public health challenge [22] due to the limitation in the therapeutic options for those strains. Unfortunately, this crisis has grown until the appearance of both XDR and pandrug-resistant (PDR) strains [23]. Food-borne diseases caused by resistant non-typhoid salmonella are considered important public health threats worldwide [24]. Several reports tackle the problem of MDR foodborne infections by renewal of the therapeutic options of the medicinal plants [25] or drug repurposing [26]. Essential oils extracted from the leaves of *Paramignya trimera* and *Limnocitrus littoralis* have antibacterial, antiviral, antimycotic, and antitrichomonas effects [27]. In the same context, Monoterpenes and sesquiterpenes essential oils extracted from *Aloysia, Lantana, Lippia, phyla,* and *Stachytarpheta* genera as well as *L. camara*, *C. citriodora,* and *Austroeupatorium inulaefolium* have been found to possess synergistic antimicrobial activities with other antibiotics [28,29,30]. Of note, both thymol and thyme essential oils were used as promising antibacterial, antiviral, antiseptic, and anti-inflammatory agents. Additionally, they have antioxidant, anticarcinogenesis, and antispasmodic properties. Surprisingly, novel studies have reported their antibiofilm, antifungal, and antileishmanial activities [31,32]. Unfortunately, there are very scarce studies on fighting XDR *S.* Enteritidis infections. In this context, our report highlighted the use of thymol nanoemulsion as an antimicrobial, anti-virulence, and immunostimulant agent for controlling this type of infection.

Multidrug-resistant phenotypes described among *S.* Enteritidis were recorded worldwide as well as in our report, which was indicated by the high prevalence of an MDR pattern among our strains (83.9%). There is an increase in the drug resistance among *S.* Enteritidis strains [33]. Fortunately, and in accordance with our study, a low prevalence rate of XDR *S*. entritidis was detected [34].

In this work, thymol essential oil was formulated as a water-dispersible nanoemulsion. In agreement with a previous report, the antibacterial activities of thymol and its derivatives were confirmed against important food-borne pathogen [35]. Herein, the bacteriostatic and bactericidal activities of thymol nanoemulsion against both MDR and XDR *S.* Enteritidis strains were detected in vitro by measuring MIC and MBC values. Additionally, the reduction in the DNA copies of the *sefA* gene observed during the in vivo studying of the thymol nanoemulsion effect on XDR *S.* Enteritidis confirmed its antimicrobial activities. It was postulated that the antimicrobial mode of action of thymol is disrupting the cells membrane leading to a rapid leakage of the intracellular components [36]. This finding was confirmed in our study, where the antimicrobial activity of thymol nanoemulsion was detected by observing the ultrastructure of treated *S.* Enteritidis strains under TEM. The treated strains showed a complete destruction of the cells, thickening and damage of both the cell wall and cell membrane, and cytoplasmic leakage and clumping.

*Bifidobacterium* sp. is one of the gut microbiome, which plays important roles through controlling the immune system [37]. Additionally, it has an inhibitory action against Gram negative and positive pathogens and saprophytes through the production of microbial proteinaceous compounds [38]. It was documented in a previous study, as well as in our study, that the essential oils have flourish effects on the bifidobacteria and other microbiotas [39]. The increasing in the number of bifidobacteria in addition to the direct bactericidal effect of thymol nanoemulsion may illustrate the decreasing in the *Salmonella* sp. counts in the challenged broilers. The powerful antibacterial activity of thymol nanoemulsion may be attributed to the fact that nanodroplets can easily penetrate and directly disrupt the bacterial membranes [40].

Recently, many therapeutic options targeting bacterial virulence rather than bacterial survivals have been released [41]. For that, the anti-virulence activity of thymol nanoemulsion was assessed through the detection of *sopB* gene expression in response to thymol nanoemulsion treatment. Our finding reported that the *sopB* gene expression was sharply decreased in the challenged chicken groups treated with 1.5 and 2.25% thymol nanoemulsion. This notable finding was ascertained by the pronounced improvement in the body weight gain and feed conversion ratio as well as decreases in the severity of clinical findings and mortality percentages, especially in the challenged group treated with 2.25% thymol nanoemulsion. In accordance with our study, a reduced mortality rate and an improved growth gain and feed conversion ratio were observed upon supplementation of chicken diet with microencapsulated blends of natural identical essential oils especially thymol [42]. Our present in vivo observations parallel the previous study, which confirmed the anti-virulence activities of thymol oil and thymol nanoemulsion against different *Salmonella* species [43]. The anti-virulence activities of thymol nanoemulsion may be attributed to its quorum sensing (QS) inhibitory actions. A previous study reported that the essential oils inhibit QS at sub-MICs and attenuate a variety of QS indicators in a dose-dependent manner [44]. The anti-QS activities of thymol essential oil may be attributed to their direct action on QS signaling molecules synthesis and the inactivation of cognate receptors. Therefore, the expression of virulence genes necessary for cooperative behaviors is inhibited [41].

One of the most important strategies to prevent the infection with resistant pathogens is increasing the strength of the immune systems [45,46]. Interestingly, thymol has been pronounced to have a potent immunostimulating activity with respect to humoral and cellular immunity [47]. The immunomodulatory and anti-inflammatory activities of thymol essential oil in addition to their mechanisms were discussed previously [39]. Thymol and its derivatives have been reported to exert their immunomodulatory activities through the modulation of T cell activity by decreasing IL-2 and IFN-γ production, possibly through down regulation of activating protein [AP-1] and nuclear factor of activated T cells [NFAT-2] transcription factors [48]. In another report, thyme extract showed significant anti-inflammatory properties as it modulated the release of IL-1β and IL-8 and down-regulated NF-κB p65 and NF-κB p52 proteins [49]. Accordingly, thymol nanoemulsion proved to have protective effects in our study in a dose-dependent manner through the up-regulations of multiple proinflammatory cytokine genes; *IL1β*, *IL12α*, *IFN-γ*, and *TNF-α*, which reached a steady state after treatment with 2.25% thymol nanoemulsion. Meanwhile, our results revealed reductions in the expression of other cytokine genes; *IL-4* and *IL-10*, which inhibit host defenses against salmonella infections [10].

## 4. Material and Methods

### 4.1. Ethical Statement

In this study, 12 and 19 *S.* Enteritidis strains were isolated from 150 chicken meat and 250 stool samples of diarrheic patients, respectively. All human samples were obtained with the sole aim to care for patients by proper diagnosis and treatment. Therefore, the ethical approval of participants was not necessary as all clinical and laboratory data were obscured. The care and management of experimental birds were in accordance with guidelines of Institutional Animal Care and Use committee of Faculty of Veterinary Medicine at Zagazig University (ZU-IACUC/2/F/16/2020).

### 4.2. Thymol Nanoemulsion Characterization

The thymol nanoemulsion was kindly provided from the National Center for Radiation Research and Technology (NCRRT), Atomic Energy Authority, Egypt. The size, morphology, and stability of the provided thymol nanoemulsion were characterized using Zeta potential measurements and TEM.

### 4.3. Identification of MDR and XDR S. Enteritidis Strains

All 31 *S.* Enteritidis strains were confirmed phenotypically based on standard bacteriological methods, serotyping technique including slide and tube agglutination tests and genotypically depending on PCR analysis of the *sefA* gene [4]. The antimicrobial susceptibility of the identified *S.* Enteritidis strains was detected using the Kirby–Bauer disk diffusion method. Strains susceptibility was tested against 16 antimicrobials (Oxoid, Basingstoke, UK) representing different categories indicated for *Enterobacteriaceae* [23] including ampicillin (AMP, 10 µg), amoxicillin/clavulanic acid (AMC, 20/10 μg), cefoxitin (FOX, 30 μg), gentamicin (CN, 10 μg), ceftriaxone (CRO, 30 μg), chloramphenicol (C, 30 μg), piperacillin/tazobactam (TZP, 110 μg), cefazoline (CZ, 30 μg), fosfomycin (FOS, 50 μg), tigecycline (TGC, 15 μg), aztreonam (ATM, 30 μg), sulfamethoxazole/trimethoprim (SXT, 25 μg), ciprofloxacin (CIP, 5 μg), tetracycline (TE, 30 μg), colistin sulfate (CT, 10 μg), and imipenem (IPM, 10 μg). The results of the disc diffusion test were confirmed by estimating the MIC values using microdilution method [50]. The MDR strains were defined as those which were resistant to three or more different antimicrobial classes. Meanwhile, the strains remained susceptible to only one or two from all of the tested antimicrobial categories that were considered as XDR [23].

### 4.4. Determination of Thymol Nanoemulsion MIC and MBC Values

The MIC of thymol nanoemulsion was investigated against MDR and XDR strains using the broth microdilution method. Aliquots of thymol nanoemulsion were diluted in 96-well microtiter plates containing Mueller Hinton broth (MHB, Oxoid, Basingstoke, UK) medium to produce a range of concentrations from 0.01 to 15% (*v*/*v*). The MIC values were defined as the lowest concentration of thymol nanoemulsion, which completely inhibited the microbial growth [50,51]. Meanwhile, the lowest concentration of thymol nanoemulsion that revealed no visible growth after sub-culturing on fresh medium was defined as MBC [52,53]. Colistin sulfate (Oxoid, Basingstoke, UK) was served as a positive control; meanwhile, sterile water was included in every plate as a negative control.

### 4.5. Ultrastructure Examination of Thymol Nanoemulsion Treated MDR and XDR S. Enteritidis Strains using TEM

The MDR and XDR *S.* Enteritidis strains were treated with the MBC concentrations of thymol nanoemulsion. The treated strains were examined by TEM (JEOLJEM-1010, JEOL Ltd., Tokyo, Japan), which was performed in the Regional Center of Mycology and Biotechnology, Al-Azhar University [54].

### 4.6. Chicken Housing, Management, and Experimental Design

A total of 360 one-day-old male Ross 308 boiler chicks purchased from a local commercial hatchery farm were used in this study. All birds were checked to be free from *S.* Enteritidis infection depending on bacteriological examination of the cloacal swabs and fecal samples. The birds were randomly assigned into six groups (three replicates/group and each replicate consisted of 20 birds). Four bird groups were challenged with identified XDR *S.* Enteritidis strain and treated different MIC values of thymol nanoemulsion. One positive control group was challenged only, while another negative control group was kept unchallenged and untreated. In agreement with Ross Broilers Management Guide, all birds were subjected to light for the first 24 h and then 23-h light/1-h dark cycle until the end of the study. An antibiotic-free, coccidiostat free, mash basal diet was prepared for starter (day 1–21) and grower (day 22–42) periods according to the nutrition specification of Ross Broiler Handbook [55]. The chemical analyses (moisture, crude protein, ether extract, and crude fiber) of all feed ingredients were assessed using the standard method as recommended by Association of Official Analytical Chemists, AOAC [56]. Birds were provided feed and water ad libitum throughout the experimental period.

#### 4.6.1. Experimental Infection by XDR *S.* Enteritidis

At 30 days old, all experimental birds with the exception of the negative control group were orally challenged with *S*. Enteritidis inoculum with a concentration of 10^8^ CFU/mL (one mL/bird) [57]. Two chickens from each challenged group were slaughtered 24-h post challenge (after clinical signs appearance) to check the infection through re-isolation and identification of the challenging strain. Moreover, re-testing of the antimicrobial susceptibility pattern was done to ensure that the recovered strain corresponded to the challenging one.

#### 4.6.2. Post Infection Treatment

At 31 days old, the birds in the four challenged groups were treated with 0.25, 0.5, 0.75, and 1 MIC values of thymol nanoemulsion in drinking water (*v*/*v*) for 5 successive days. Meanwhile, the birds in positive and negative control groups consumed untreated water.

#### 4.6.3. Treatment Evaluation Parameters

##### Clinical Examination

Chickens in all experimental groups were observed daily following *S.* Enteritidis challenge. Clinical signs, mortality, and gross lesions were recorded just after challenge until the end of the experimental period.

##### Growth Performance

Body weight of the birds in each replicate was recorded and averaged at the beginning (initial weight) and at the end (final weight) of the experiment. The average body weight gain and the feed conversion ratio in all experimental chicken groups were then calculated.

### 4.7. Microbiological Analyses

After the end of the treatment, all birds were slaughtered and homogenized cecal contents were 10-fold diluted in sterile phosphate-buffered saline. Total aerobic bacterial counts were measured on Standard Methods Agar (Oxoid, Basingstoke, UK) plates following aerobic incubation at 37 °C for 2–3 days. Total bifidobacterium counts were determined on M-MRS (Maltose-deMan Rogosa Sharpe) agar medium (Oxoid, Basingstoke, UK) plates after anaerobic incubation at 37 °C for 3 days. Salmonella counts were also detected on xylose lysine deoxycholate agar (Oxoid, Basingstoke, UK) after incubation at 37 °C for 24 h. The average results of the triplicated measurements were determined as log_10_ colony forming units (CFU)/g of the cecal contents.

### 4.8. Quantification of S. Enteritidis DNA Copies of sefA Gene

The QIAamp DNA Stool Mini Kit (Qiagen GmbH, Hilden, Germany) was used to extract DNA from the cecal samples of slaughtered birds according to the supplier’s recommendations. Extracted DNA concentrations and quality were measured spectrophotometrically with a Spectrostar NanoDropTM 2000 spectrophotometer (Thermo Fisher Scientific Inc., Waltham, MA, USA). Purified DNA was stored at −80 °C for the subsequent quantitative PCR analysis. A real time PCR (RT-PCR) assay was used to determine salmonella counts in cecal samples. The PCR primer and TaqMan probe sets targeting the *sefA* gene, which encodes *Salmonella* Enteritidis fimbrial protein were used as described previously [58]. The salmonella concentrations were measured by interpolating the Ct values of DNA samples into the generated standard calibration curves and then their log_10_ of the CFU numbers were calculated.

### 4.9. Gene Expression Analysis of Salmonella SopB and Cytokines

The QIAamp RNeasy Mini kit (Qiagen GmbH, Hilden, Germany) was used to obtain purified RNA from chicken cecal and splenic samples. The concentration of the extracted RNA was measured using a Spectrostar NanoDropTM 2000 spectrophotometer (Thermo Fisher Scientific Inc., Waltham, MA, USA). The expression levels of genes encoding SopB and IFN-γ, TNF-α, IL-1β, IL-12α, IL-4, and IL-10 were detected by one step RT-qPCR assay using Qiagen QuantiTect Probe RT-PCR kit (Qiagen, Inc., Valencia, CA, USA) on a Stratagene MX3005P real time PCR machine (Agilent Technologies, Inc., Santa Clara, CA, USA). All PCR reactions were done in triplicate. The expression levels of *sopB* gene was normalized using *16S rRNA* as an internal housekeeping gene, while that of cytokine genes were assessed using *28S rRNA* as an endogenous reference gene. The relative gene expression data were assessed using the 2^−ΔΔCt^ method [59]. Target gene primers and probes used in this study have been described previously [60,61,62,63].

### 4.10. Statistical Analysis

To determine if there is a significant difference among groups (*n* = 6), one-way ANOVA was run in triplicate and *p*-values < 0.05 were considered as a cutoff for significance. F value for one-way ANOVA analysis was estimated. The analyses was done using graph Pad prism software version 8. Author/Product: GraphPad Software/GraphPad Prism. https://www.filehorse.com/download-graphpad-prism/.

## 5. Conclusions

Thymol nanoemulsion was recognized as a promising, safe, and alternative therapeutic option in treating both MDR and XDR *S.* Enteritidis with notable anti-virulence activities, which hinder the pathogenic pathway. From another point of view, it had immunostimulant activities, which increase the possibility to be used in non-bacterial infections. So, we highly recommended the use of thymol nanoemusion as a feed additive, especially for immunocompromised patients, animals, and chickens for those that are at a higher risk of infection. Additionally, the results of our study contribute towards correct therapeutic decision-making and offer a new therapeutic option for the resistant foodborne pathogens. We hope that further studies will formulate the thymol nanoemulsion alone, or with other antimicrobial drugs in suitable pharmaceutical forms or as feed additives, and approve it for medical usage.

## Figures and Tables

**Table 1:** Effects of thymol nanoemulsion therapeutic supplementation on growth performance and cumulative mortality of broiler chickens post challenge with XDR *S.* Enteritidis strain.

Group		Initial Body Weight	Final Body Weight	Final Body Weight Gain	Feed Conversion Ratio	Cumulative Mortality Percentage
		(g/bird)	(g/bird)	(g/bird)		
Control	Negative	43	2409	2366	1.75	3
	Positive	46	1782	1736	2.42	22
Challenged groups treated with different doses of thymol nanoemulsion (%)	0.75	40	2012	1972	1.93	12
	1.5	50	2082	2032	1.95	10
	2.25	39	2397	2358	1.67	5
	3	35	2279	2244	1.87	6
*p*-value		0.0005	<0.0001	<0.0001	<0.0001	<0.0001
F value		10.49	3015	4113	73.73	50.26

## Data Availability

All data generated or analysed during this study are included in the published article or as supplementary information files and will be available after article publication.

## Supplementary Materials

The following are available online at https://www.mdpi.com/2079-6382/10/1/25/s1, Table S1: MIC and MBC values (%) of thymol nanoemulsion against MDR and XDR *S*. Enteritidis strains.
