# Supplementary material for: Thymol Nanoemulsion: A New Therapeutic Option for Extensively Drug Resistant Foodborne Pathogens
Source: Antibiotics (Basel). 2020 Dec 30;10(1):25. doi: 10.3390/antibiotics10010025 (PMC7823989; doi:10.3390/antibiotics10010025)
Supplement: Supplementary file 1 [file antibiotics-10-00025-s001.pdf]

Table S1. MIC and MBC values (%) of thymol nanoemulsion against MDR and XDR *S*. enteritidis strains.

Strain Resistance Category	Strain Code	MIC (% v/v)	MBC (% v/v)
MDR	1	0.5	1
	2	1	1
	3	1	2
	4	0.5	1
	5	1	2
	6	1	2
	7	1	2
	8	0.5	2
	9	1	2
	10	0.5	1
	11	1	2
	12	1	2
	13	1	2
	14	1	2
	15	0.5	1
	16	0.5	1
	17	0.5	1
	18	1	2
	19	1	2
	20	1	2
	21	0.5	2
	22	1	2
	23	0.5	1
	24	1	2
	25	1	2
	26	1	2
	27	0.5	1
	28	1	2
	29	1	2
	30	1	2
**XDR**	**31**	**3**	**5**

MDR: multidrug resistant, XDR: extensively drug resistant, MIC: minimum inhibitory concentration, MBC: minimum bactericidal concentration.
